# Diglossia and Orthographic Complexity as Multiplicative but not Additive Challenges in Arabic: A Critical Review

**DOI:** 10.1007/s10936-026-10214-3

**Published:** 2026-04-04

**Authors:** Ibrahim Asadi, Abeer Asli-Badarneh

**Affiliations:** 1 Department of Learning Disabilities and Special Education, The ArabAcademic College for Education, 22 Hahashmal St, P.O. Box 8340, Haifa, Israel; 2 The Unit for the Study of Arabic Language, Edmond J. Safra Brain Research Center for the Study of Learning Disabilities, Faculty of Education, University of Haifa, Haifa, Israel

**Keywords:** Arabic, Diglossia, Literacy, Multiplicative challenges, Orthography

## Abstract

The present review explores how the unique linguistic and orthographic characteristics of Arabic interact to shape the development of literacy in Arabic. The review is based on a systematic synthesis of empirical studies published between 2000 and 2025, including behavioral, developmental, and neurocognitive research on reading, spelling, and comprehension among native Arabic speaking learners, conducted in line with PRISMA guidelines. It focuses on the combined influence of diglossia and the visual and structural complexity of the Arabic script. Drawing on empirical findings from behavioral, developmental, and neurocognitive research, the review shows that these two dimensions do not merely add their effects but interact in a multiplicative way, creating cumulative challenges for beginning readers and for those with reading difficulties. Across the studies reviewed, a consistent pattern emerges showing that this interaction constrains decoding accuracy, reading fluency, and comprehension across developmental stages. These effects are particularly evident in the early phases of literacy acquisition and are amplified among learners with dyslexia or developmental language disorder. The discussion critically examines how universal models of reading fail to account for these interacting sources of difficulty, and argues for a language-specific framework that captures the dynamic relation between phonological, morphological, and orthographic processes in Arabic. From an instructional perspective, the synthesis points to the need for teaching practices that explicitly bridge spoken and standard varieties of Arabic, place greater emphasis on morphological awareness, and provide careful scaffolding as learners transition from vowelized to unvowelized text. The review concludes with implications for theory and pedagogy, emphasizing the need for instructional practices that bridge spoken and standard Arabic.

## Introduction

Reading is a cornerstone of education and social life (OECD, 2019). Unlike spoken language, which develops naturally, reading requires coordination of visual, phonological, orthographic, and semantic systems (Perfetti, 2007). While these have been well studied in alphabetic scripts, Arabic presents unique challenges that call for rethinking how literacy theories account for complex writing systems (Taha, 2013).

## Theoretical Frameworks and Their Limits

Several models have guided explanations of reading development. The Dual-Route Model distinguishes between lexical and phonological routes to word recognition (Coltheart, 2005; Zorzi, 2010), while the Lexical Quality Hypothesis emphasizes the integration of phonological, morphological, and semantic representations for efficient word identification (Perfetti, 2007). The Simple View of Reading links comprehension to the combined effects of decoding and oral language skills (Gough & Tunmer, 1986). Though influential, these frameworks assume a close correspondence between spoken and written language, transparent grapheme–phoneme mappings, and stable lexical representations—conditions not met in Arabic. Consequently, their explanatory power is limited, and they require adaptation to account for the linguistic and orthographic realities of Arabic.

### The Arabic Challenge: Diglossia and Orthographic Complexity

Arabic presents two core challenges for literacy models. The first is diglossia, a sociolinguistic divide between Spoken Arabic (SpA), acquired naturally at home, and Standard Arabic (StA), learned formally and used in education and print. Readers must process texts in a linguistic system distinct from their daily speech across phonological, morphological, lexical, and syntactic levels (Ferguson, 1959; Saiegh-Haddad & Henkin-Roitfarb, 2014). The second challenge is orthographic. Arabic’s 28 consonantal letters vary by position (initial, medial, final, isolated), and as an *abjad*, it omits short vowels, creating an opaque script that requires reliance on context for meaning (Taha, 2013). Moreover, its root-and-pattern morphology links orthography and morphology (Asadi et al., 2023), making morphological awareness essential for fluent reading (Asadi et al., 2024; Asli-Badarneh et al., 2022; Asli-Badarneh & Leikin, 2019; Boudelaa & Marslen-Wilson, 2013).

### Limitations of Existing Models

The MAWRID model (Saiegh-Haddad, 2018) remains the only comprehensive framework addressing Arabic word reading, highlighting vowelization, morphology, and diglossia as central components. Yet it does not fully explain how diglossic distance and orthographic opacity interact across development or learner populations. Neurocognitive studies indicate that processing demands vary by dialect and emerge early in ERP responses (Andria et al., 2022), while developmental research shows that lexical distance effects—whether words are identical, cognate, or unique, persist or even intensify with age (Asli-Badarneh & Asadi, 2023). These findings underscore the need to refine both universal and Arabic-specific models, calling for an integrated framework that captures the dynamic interplay between linguistic distance and orthographic complexity.

### Methodological Limitations in Current Research

Most studies on Arabic reading have examined phonological awareness, word and nonword reading, fluency, and comprehension (Saiegh-Haddad & Everatt, 2017; Schiff & Saiegh-Haddad, 2017), but they rely on small, tightly controlled tasks that reveal processes without reflecting real diglossic reading (Eviatar & Ibrahim, 2014). Few have compared performance on StA and SpA texts, despite evidence that word familiarity affects reading efficiency (Asaad & Eviatar, 2013; Saiegh-Haddad & Schiff, 2016). These gaps have consequences: Arabic-speaking students score below international literacy benchmarks such as PIRLS (Saiegh-Haddad & Joshi, 2014), and difficulties are especially pronounced, and understudied, among children with dyslexia or developmental language disorder (Saiegh-Haddad & Ghawi-Dakwar, 2017; Schiff & Saiegh-Haddad, 2017).

### The Diglossic Challenge: Linguistic Distance and Literacy Implications

Arabic is a clear case of diglossia: Children acquire SpA naturally, while StA is formally taught (Ferguson, 1959). The two differ in phonology, morphology, lexicon, and syntax (Saiegh-Haddad, 2018), with variation across dialects and lexical domains (Watson, 2011). Corpus studies show that only 21% of Palestinian Arabic words are identical across varieties (Saiegh-Haddad & Spolsky, 2014), while over 60% of children’s speech uses SpA forms (Asli-Badarneh et al., 2023), limiting exposure to the language of literacy. Yet corpus data show what differs, not how it affects processing, highlighting the need for empirical work.

Experimental evidence shows that diglossia carries educational costs: Preschoolers identify StA phonemes less accurately (Saiegh-Haddad et al., 2011), and reduced fluency persists into adolescence (Saiegh-Haddad & Schiff, 2016). It also hinders word learning in children with developmental language disorder (Ghawi-Dakwar & Saiegh-Haddad, 2025), constrains narrative (Tallas-Mahajna et al., 2022), comprehension and academic literacy (Poyas & Bawardi, 2018) and even social emotional learning (Asli-Badarneh, 2025). Research still focuses mainly on phonological and lexical distance, leaving morphosyntactic and discourse-level aspects underexplored (Saiegh-Haddad & Armon-Lotem, 2025; Saiegh-Haddad & Schiff, 2016, 2025). Processing effort also varies by dialect, as shown in ERP and behavioral studies (Andria et al., 2022).

There is no single “diglossic effect”. Its form varies by word type (Asadi & Asli-Badarneh, 2023), linguistic domain, dialect (Watson, 2011), and learner profile (Ghawi-Dakwar & Saiegh-Haddad, 2025). These interacting factors explain cross-study variability and call for models that capture this complexity. Existing frameworks often treat diglossia mainly as a phonological or lexical issue, overlooking its wider linguistic and educational dimensions (Saiegh-Haddad & Armon-Lotem, 2025).

## Orthographic Complexity: Beyond Surface-Level Challenges

In addition to diglossia, Arabic orthography introduces unique difficulties. Unlike alphabetic systems with stable grapheme-phoneme correspondences, Arabic integrates visual, phonological, and morphological features that interact in complex ways. Orthographic complexity, therefore, involves several intertwined challenges. The script’s 28 consonantal letters change shape according to position (initial, medial, final, or isolated) and six letters (ا، د، ذ، ر، ز، و) never connect to the following one, creating segmentation breaks that confuse beginners (Taha, 2013). Many letters differ only by the number or placement of dots (ب، ت، ث), leading to visual confusions (Taha, 2016). Learners must recognize that multiple forms represent the same phoneme, requiring mastery of positional allography (Saiegh-Haddad & Henkin-Roitfarb, 2014). The script’s cursive nature adds another layer: while skilled readers may benefit from connected letter flow, connectivity can hinder struggling readers or those with dyslexia (Khateb et al., 2014). Findings remain mixed, reflecting methodological differences and individual variation in reading profiles.

Second, Arabic’s abjad script omits short vowels, creating orthographic opacity that forces readers to rely on morpho-syntactic and semantic cues to interpret homographs. Vowelization is inconsistently applied, fully in primers, partially in textbooks, and absent in most print, leading to uneven developmental progress and reducing comparability across educational contexts (Abu-Rabia, 2007).

The root–pattern system both supports and challenges reading development. Consonantal roots group words into lexical families, fostering vocabulary growth and morphological awareness. However, young readers must learn to segment and reconstruct these structures, an especially difficult task in unvoweled text (Boudelaa & Marslen-Wilson, 2013). Current models rarely clarify when morphology facilitates reading and when it hinders it, leaving its developmental role only partly understood.

Around fourth grade, vowelization marks are gradually removed, making Arabic script less transparent and forcing readers to depend more on morphological and lexical knowledge (Saiegh-Haddad & Henkin-Roitfarb, 2014). However, few longitudinal studies have examined how children adapt to this shift. Existing cross-sectional evidence points to temporary setbacks and periods of reorganization but provides only a partial understanding of the developmental process (Abu-Rabia, 2007).

Finally, Children’s dialectal backgrounds strongly influence how they engage with Arabic script. Spoken varieties differ in phonology, morphology, and vocabulary, affecting how readily readers can align dialectal speech with the Standard Arabic orthography (Watson, 2011). Although this interaction is recognized, it has rarely been studied systematically. As a result, “orthographic opacity” likely reflects overlapping challenges that shift with development, linguistic background, and educational context (Eviatar & Ibrahim, 2014; Saiegh-Haddad & Henkin-Roitfarb, 2014).

Research on Arabic reading remains methodologically constrained. Most studies use single-word tasks that neglect the linguistic and cognitive demands of connected text, and even text-based studies emphasize speed over comprehension or strategy use (Saiegh-Haddad & Schiff, 2025; Schiff & Saiegh-Haddad, 2017). The lack of longitudinal research leaves open how children adapt to unvoweled print or how vowelization and connectivity interact over time, contributing to inconsistent findings. Ultimately, Arabic readers face a dual challenge—learning to read in Standard Arabic, distinct from their spoken dialect, while mastering a visually complex and partly opaque script. Given the internal variability of both script and dialect, research has only begun to address how these interacting factors shape literacy development (Eviatar & Ibrahim, 2014; Saiegh-Haddad & Henkin-Roitfarb, 2014).

## The Interactive Challenge: Multiplicative Effects

When diglossia and orthographic complexity intersect, the difficulty multiplies. StA differs from the spoken dialect in phonology and morphology, requiring readers to decode an opaque script while processing unfamiliar forms. Asaad and Eviatar (2013) showed that opacity affects Standard Arabic–unique words more than shared ones, proving that both factors reinforce each other. Still, most studies rely on controlled word lists with limited ecological validity, and results are mixed, some show broad effects on fluency and accuracy, others only on specific skills (Saiegh-Haddad & Schiff, 2016, 2025).

Variation among learners further complicates the picture. Children exposed to it outside school or growing up multilingual often follow distinct literacy paths (Saiegh-Haddad & Schiff, 2025). These differences underscore the role of sociolinguistic context: variations in literacy experience, home language use, socioeconomic background, and multilingual proficiency all influence how children manage the dual demands of reading and understanding Arabic.

The combined effects of diglossia and orthographic opacity are most pronounced in early schooling but change with increased exposure to Standard Arabic and unvoweled text—sometimes easing, sometimes shifting (Asadi et al., 2023). However, the lack of longitudinal research leaves this progression unclear. Most existing studies rely on controlled word-reading tasks that isolate variables but shed little light on how children process connected text. Research on extended reading, comprehension, and higher-order skills such as inference or academic literacy remains limited (Saiegh-Haddad & Schiff, 2025).

## Vulnerable Populations and Theoretical Gaps

Children with developmental language disorder (DLD) or dyslexia experience intensified challenges from both diglossia and orthographic opacity (Schiff & Saiegh-Haddad, 2017, 2018). Electrophysiological evidence shows atypical conflict monitoring during language processing (Froud & Khamis-Dakwar, 2021), yet these groups remain understudied, reducing ecological validity. Phonological deficits interact with script opacity, increasing the risk of persistent reading difficulties (Ghawi-Dakwar & Saiegh-Haddad, 2025; Schiff & Saiegh-Haddad, 2018).

Existing frameworks, including MAWRID (Saiegh-Haddad, 2018), address vowelization, morphology, and diglossia but neglect their developmental interaction. A broader, context-sensitive model integrating cross-sectional and longitudinal findings is needed.

Educationally, Arabic-speaking students score below international benchmarks such as PIRLS, reflecting the combined impact of diglossic and orthographic barriers (Saiegh-Haddad & Joshi, 2014). These challenges extend beyond decoding to comprehension and academic literacy (Saiegh-Haddad & Schiff, 2016, 2025). Morphological awareness—central to the root–pattern system—remains underemphasized (Saiegh-Haddad & Taha, 2017), while neurocognitive findings reveal distinct reading mechanisms (Andria et al., 2022). Vulnerable learners are most affected, highlighting the need for inclusive, context-aware instruction that addresses both linguistic and orthographic complexity.

## Present Review Objectives

This review moves beyond descriptive accounts of Arabic literacy and takes a critical look at how diglossia and orthographic complexity together constrain reading development. It highlights three key themes: the internal diversity of these constructs, the methodological limitations that have shaped prior research, and the educational stakes of the unresolved gaps. Building on these concerns, the review is guided by four interrelated questions:

1. **Linguistic distance and foundational processes**
  In what ways does the gap between SpA and StA affect phonological awareness, morphological awareness, syntax word recognition, fluency, and comprehension?
2. **Orthographic sources of difficulty**
  Which features of the Arabic script, such as letterform variation, cursive connectivity, vowelization practices, and the root–pattern system—pose the greatest obstacles to decoding and comprehension, and how do their effects shift across developmental stages?
3. **Interactive effects**
  Do diglossic and orthographic factors act separately, or do they combine in multiplicative ways? And are these effects consistent across different tasks and age groups?
4. **Populations**
  How are these challenges experienced by learners with diverse profiles, including children with dyslexia, developmental language disorder, or other vulnerabilities?

The findings from these questions are synthesized to examine broader theoretical implications. The review evaluates how established models, the Dual-Route Model, the Lexical Quality Hypothesis, and Arabic-specific frameworks like MAWRID, must be reconsidered in view of the multiplicative effects observed in Arabic literacy development. This synthesis forms the basis for the Discussion, which proposes a more comprehensive and context-sensitive framework.

## Methods

### Search Strategy

This review followed PRISMA guidelines (Page et al., 2021) and targeted empirical studies published between January 2000 and July 2025 examining the effects of diglossia and orthographic complexity on literacy development in native Arabic-speaking children and adolescents. The choice of this time frame reflects developments in the study of Arabic literacy. From the early 2000s onward, research on diglossia and orthographic complexity increasingly adopted empirical, cognitively grounded approaches. In addition, this period allows the review to capture longitudinal trends in the literature, as studies progressively expanded in terms of age groups, literacy outcomes, and learner populations, enabling a synthesis that reflects both developmental patterns and shifts in theoretical perspectives over time. A comprehensive search was conducted across PubMed Central, PsycINFO, ERIC, LLBA, Web of Science, Frontiers, and Google Scholar. Search terms covered four key domains: (1) literacy outcomes, (2) phonological and lexical distance, (3) orthographic complexity, and (4) diglossia and linguistic variation.

Keyword combinations included: *“diglossia,” “spoken Arabic,” “Modern Standard Arabic,” “colloquial Arabic,” “lexical distance,” “phonological novelty,” “cognate,” “visual-orthographic complexity,” “vowelization,” “Arabic script,” “reading,” “spelling,” “decoding,” “dyslexic”* and *“comprehension.”* Boolean operators were applied systematically (*AND* between domains, *OR* within domains), and queries were adapted to each database’s syntax. Only peer-reviewed, English-language articles were included, regardless of country of origin.

### Selection Process

**Fig. 1:**
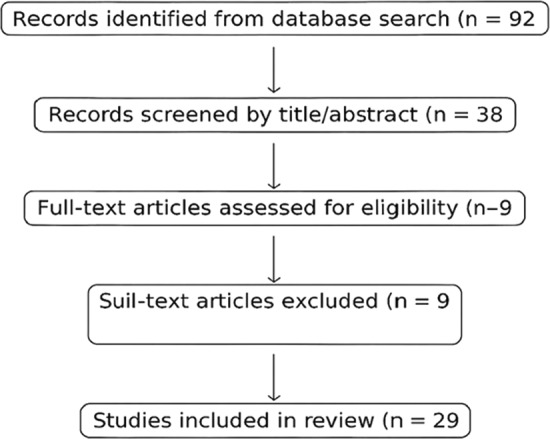
Shows a flowchart of each phase in this procedure in the PRISMA manner

The combined search produced 92 unique records after duplicates were removed. Titles and abstracts were screened for relevance to the review’s guiding questions, leading to the exclusion of clearly unrelated studies. Thirty-eight full-text articles were then examined in detail against predefined inclusion and exclusion criteria. Of these, 29 met all eligibility requirements and were included in the final synthesis (see Appendix). Selection prioritized methodological rigor and developmental representativeness across age groups, dialects, and assessment types. The process is summarized in Fig. 1 (PRISMA flow diagram).

### Inclusion and Exclusion Criteria

Studies were included if they examined monolingual first-language Arabic speakers and addressed diglossic variation, orthographic complexity, or both in relation to reading, spelling, or comprehension. Eligible designs included experimental, quasi-experimental, or longitudinal studies that reported empirical findings on Arabic script literacy. Studies were excluded if they focused on bilinguals or L2 learners, treated diglossia only theoretically, lacked empirical data, or used non-Arabic scripts (e.g., Latin, Hebrew, Romanized Arabic).

Quality standards required that studies (a) addressed the review’s core topic, (b) described literacy tasks in detail, and (c) clearly reported participant characteristics (age, dialect, proficiency, diagnostic status). All included studies were peer-reviewed. Dialectal affiliation was noted to assess generalizability, and studies with unclear design, missing participant details, or inadequate methodological reporting were excluded. Only research directly illuminating how diglossia or orthography shapes Arabic literacy was retained (See Table 1 for the included studies).

### Data Extraction and Classification

Data were extracted using a structured template recording authorship, year, participant characteristics (age, dialect, developmental status), research focus (orthography, diglossia, or both), task type (e.g., RAN, spelling, comprehension), manipulated variables (e.g., vowelization, lexical overlap), outcome measures (accuracy, fluency, errors), and key findings.

Studies were categorized into four themes: Linguistic distance and foundational processes, Orthographic complexity (letterform variation, vowelization, visual load), Interactive effects between linguistic and orthographic challenges, Populations (learner diversity and developmental profiles).

This deductive framework, informed by the Dual-Route Model, the Lexical Quality Hypothesis, and the Linguistic Distance Hypothesis, allowed systematic comparison while accounting for methodological variation.

The identification of the four main themes was guided by a theory informed, data-driven process. Specifically, the thematic structure was developed through an iterative synthesis of the reviewed literature, informed by established theoretical frameworks in reading research, including the Dual-Route Model, the Lexical Quality Hypothesis, and Arabic-specific models such as MAWRID. Initial categories were derived from recurrent constructs and outcomes examined across studies (e.g., linguistic distance, orthographic features, and learner profiles). These categories were then refined through comparative analysis to capture broader patterns of convergence, resulting in four overarching themes that reflect both theoretical relevance and empirical consistency across the reviewed research.

## Results

Findings are organized around four key research questions examining how diglossia and orthographic complexity interact in shaping Arabic literacy development. Across the 29 studies reviewed, consistent patterns reveal the multiplicative nature of these challenges and their varying effects across developmental stages and learner populations.

## Linguistic Distance and Foundational Processes

The structural divide between SpA and StA constrains early reading development across multiple linguistic domains. The evidence shows that diglossic variation influences the growth of phonological, lexical, and working-memory skills in ways not accounted for by dominant reading models.

### Phonological Processing Constraints

Diglossic distance hinders phonological awareness from the earliest stages. Saiegh-Haddad (2003) showed that kindergarten and first-grade pupils struggled with StA-specific phonemes, a gap persisting through Grade 6 (Saiegh-Haddad & Haj, 2018). Longitudinal data (Asadi & Abu-Rabia, 2021) confirmed greater accuracy and faster naming for SpA items, with word length compounding difficulty. Neurocognitive findings (Khamis-Dakwar & Froud, 2007) revealed distinct processing of SpA and StA, resembling bilingual code-switching, while Tarabeh et al. (2025) showed that diglossic distance taxes working memory. Overall, diglossia limits phonological accuracy, speed, and memory, indicating that phonological instruction must deliberately bridge the spoken–standard divide.

### Word Recognition and Fluency Effects

Asli-Badarneh and Asadi (2023) found a clear lexical gradient: identical words were read most fluently, cognates moderately, and unique Standard Arabic (StA) words least efficiently. These gaps widened between Grades 1 and 2, indicating that linguistic distance increases with text complexity. In connected reading, repeated exposure to distant StA forms causes cumulative fluency delays. Similar results appear in passage reading—texts rich in identical forms improve fluency, while StA-dominant and unvoweled texts slow readers due to greater phonological ambiguity (Asaad & Eviatar, 2013). Thus, word recognition depends on lexical distance, requiring instruction that explicitly bridges spoken and standard varieties.

### Comprehension and Meaning Construction

Linguistic distance affects both decoding and comprehension. Abu-Rabia (2000) found that children with limited exposure to StA struggled most with inferential understanding, showing that diglossia constrains comprehension from the start. These challenges reflect differences in meaning construction, as weak StA representations hinder integration across phonological, orthographic, and semantic levels (Lexical Quality Hypothesis).

Intervention studies support this: Shahbari-kassem et al. (2024) showed that children comprehended stories best in the spoken variety, while scaffolded exposure to StA improved but did not eliminate difficulties. Some evidence, however, suggests partial facilitation—Asadi and Ibrahim (2014) found better performance on StA items in deletion tasks, and Saiegh-Haddad (2005) linked StA phoneme isolation to reading fluency. Longitudinal work shows that diglossic disadvantages lessen in later grades (Saiegh-Haddad & Haj, 2018; Schiff & Saiegh-Haddad, 2018), though gaps in morphological awareness persist.

Comprehension difficulties in StA extend beyond vocabulary to inference-making and text integration. Because limited exposure weakens vocabulary and listening skills, instruction should combine linguistic bridging with explicit strategies for managing the cognitive demands of shifting between spoken and standard forms.

### Morphological Processing Challenges

The morphological divide between SpA and StA poses a major obstacle to literacy, given the centrality of the root–pattern system. Joubran-Awadie and Shalhoub-Awwad (2023) showed that over 80% of morphemes differ between the two varieties, especially in verb inflection, forcing children to confront unfamiliar StA forms that differ structurally from those in their spoken dialect. Arabic’s non-linear morphology compounds the difficulty, requiring learners to manipulate root–pattern combinations under two sets of morphological rules. Asadi et al. (2024) confirmed that diglossic distance impairs morphological awareness, with StA-unique forms yielding slower and less accurate responses. Arabic reading therefore demands mastering both morphological complexity and cross-variety variation.

### Syntax Challenges

The diglossic divide in Arabic extends beyond phonology and vocabulary to syntax. Abu-Rabia et al. (2022) found that children acquire Standard Arabic syntactic structures more slowly and less consistently than those shared with their spoken dialect. This pattern shows that linguistic distance limits multiple dimensions of language competence, challenging reading models that assume a single, unified linguistic system.

## Orthographic Sources of Difficulty

Arabic orthography presents unique visual and structural challenges that evolve with development. Their impact changes as children gain reading experience and as cognitive skills mature, producing a shifting landscape of difficulty.

### Cursive Connectivity and Visual Complexity: A Developmental Paradox

A defining feature of Arabic script is its cursive structure. Unlike alphabetic systems where letters retain fixed shapes, most Arabic letters change form depending on position—initial, medial, final, or isolated. With 28 consonantal phonemes but over 100 allographic forms, children must learn that multiple shapes correspond to the same sound. For beginners, this variability and the dense connectivity of letters hinder recognition and increase visual load. Over time, however, as decoding becomes more automatic, these same features begin to support fluent word recognition, illustrating a developmental paradox: what initially impedes learning eventually facilitates efficiency.

Khateb et al. (2014) found that younger readers processed fully connected words more slowly and less accurately, but by Grade 6, connected forms were read more efficiently, and by Grade 9, connectivity effects nearly vanished, showing growing fluency. Artificially disconnected words were hardest at all ages, indicating that disrupting cursive flow impairs reading.

Arabic’s visual complexity further challenges readers: many letters differ only by dots. Abdelhadi et al. (2011) showed children read faster in Hebrew, reflecting Arabic’s heavier visual load. Within Arabic, connected forms eased processing, yet even older readers lacked a word-superiority effect, implying that high visual similarity delays lexical efficiency.

Yassin et al. (2020) reported that visual-orthographic errors—mainly letter-shape confusions and faulty ligaturing—made up over a quarter of mistakes in Grades 1–4, second only to phonological errors, underscoring the lasting influence of connectivity and allography.

Overall, dense connectivity first impedes decoding but later fosters fluent recognition, while visual similarity and reliance on diacritics continue to cause confusion. Instruction should therefore progress from partially to fully connected forms and emphasize distinctions in letter shapes, dots, and ligaturing to support mastery of Arabic orthography.

### Vowelization Regimes: Developmental Transparency Transitions

Arabic literacy shifts from fully vowelized to unvoweled print, turning reading from transparent to opaque and requiring new decoding strategies. Abu-Rabia and Hijjazi (2020) found vowelization improved comprehension in Grades 5–9, but its benefit declined with age, strongest in Grade 5, absent by Grade 7, minimal in Grade 9—indicating a transitional phase where reliance moves from diacritics to lexical and morphological cues. Effects also varied by genre, strongest for narratives and expository texts and weakest for dense morphological material like poetry. For skilled readers, diacritics hinder speed and accuracy (Taha & Azaizah-Seh, 2017). Thus, vowelization is essential early on, uneven in mid-development, and redundant later, requiring instruction matched to learners’ developmental stage and text complexity.

### Root-Pattern Morphology: Persistent Cognitive Demands

Arabic’s root–pattern system requires readers to decompose words into consonantal roots and reconstruct them using vowel and affix patterns—a demanding process that persists throughout development. Abu-Rabia (2007) found that morphological skill strongly predicted accuracy and comprehension across Grades 3, 6, 9, and 12 for both typical and dyslexic readers. For struggling readers, morphological production was decisive: without it, comprehension and recognition collapsed. Morphology in Arabic is therefore central, not supplementary, and interacts with vowelization, letter similarity, and connectivity. Effective instruction must integrate explicit morphological training with orthographic practice to build efficient reading strategies.

## Interactive Effects

When diglossia meets orthographic opacity, challenges multiply rather than merely add. Linguistic distance and script complexity reinforce one another, producing compounded effects that shape literacy development across all stages. Reading acquisition in Arabic is therefore defined by their constant interaction, influencing learners’ progress across ages, skills, and task types.

### Evidence for Multiplicative Effects Across Development

Saiegh-Haddad and Schiff (2016) examined 100 students from Grades 2–10, comparing reading of StA–unique and shared SpA words in both vowelized and unvoweled forms. Across all grades, StA words were read less accurately and fluently, and vowelization only partially eased the difficulty. Notably, the disadvantage persisted through adolescence, showing that increased exposure does not eliminate the combined effects of diglossic distance and orthographic opacity—both remain enduring constraints on Arabic reading development.

Asaad and Eviatar (2013) found in a Stroop task that first graders showed equal interference for correct and distorted words, indicating reliance on letter-by-letter decoding. By third grade, interference increased for correctly spelled words, reflecting a shift toward global word recognition. Rapid naming tasks further revealed that processing slowed for both visual and phonological neighbors, especially for letters representing sounds absent from the spoken dialect, evidence of combined orthographic and diglossic strain. Over time, diglossic effects fade, but orthographic opacity continues to affect even skilled readers. Thus, the interaction between diglossia and script complexity evolves rather than disappears, with orthography remaining the more persistent challenge.

### Interaction Effects in Early Reading Development

The joint impact of diglossia and orthographic opacity is most evident in beginning readers. Saiegh-Haddad (2005) studied first graders reading fully vowelized texts and found that, despite the script’s transparency, diglossic distance still affected performance. Phoneme isolation was significantly harder for sounds unique to StA than for those shared with SpA, and these difficulties were closely linked to slower letter recoding speed, the strongest predictor of reading fluency. This shows that even under vowelized conditions, linguistic distance continues to constrain early reading development.

Letter recoding speed reflects the interaction of several cognitive skills—rapid naming, working memory, and phoneme isolation—showing that reading outcomes arise from multiple, interdependent processes. Even with full vowelization, the burden of diglossia persists, compounded by the visual demands of Arabic script (Taha & Azaizah-Seh, 2017). Thus, phonological distance and orthographic complexity intersect at core cognitive levels, amplifying one another. Early reading therefore best illustrates these multiplicative effects, underscoring the need for instruction that targets both script-based and cognitive foundations of literacy.

### Converging Evidence from Reading Comprehension

Findings from Saiegh-Haddad and Schiff (2025) show that diglossic and orthographic factors interact strongly in shaping comprehension. Among 112 third graders, skills rooted in the spoken vernacular—morphological awareness, receptive vocabulary, and decoding fluency for SpA words, explained about 41% of the variance in StA reading comprehension, even after controlling for cognitive ability. Morphological awareness emerged as the strongest predictor, linking spoken-language competence to literacy in StA. In contrast, StA decoding accuracy and listening comprehension accounted for only modest additional variance, together bringing total explained variance to 61%, emphasizing the central role of vernacular-based linguistic skills in reading comprehension.

These findings indicate that strengthening oral and metalinguistic skills in the spoken variety can substantially improve comprehension in StA. This aligns with morpho-orthographic models such as MAWRID (Saiegh-Haddad, 2018), which highlight morphological awareness as a critical bridge in Arabic reading development. More broadly, the evidence supports an interactive view: comprehension in StA arises from the intersection of diglossia and orthographic opacity within shared linguistic and metalinguistic processes.

In short, Comprehension findings show that diglossia and orthography interact through morphological “bridges” linking spoken-language competence to Standard Arabic literacy. Pedagogically, this underscores the importance of strengthening metalinguistic skills across both varieties, rather than concentrating solely on decoding in StA.

### Task and Genre Interactions in Later Development

In adolescence, the effects of diglossia and orthographic opacity become task- and genre-dependent rather than disappearing. Abu-Liel et al. (2021) found that eighth graders read unvoweled StA faster and more accurately than vowelized text, but comprehension was higher with diacritics—indicating that decoding unvoweled words consumes resources needed for deeper understanding. In the Arabizi condition, students performed well with narratives but poorly with expository texts, showing that script familiarity aids informal reading but not academic comprehension. Thus, interactive effects persist in more differentiated forms, and instruction at this stage must extend beyond decoding to equip learners for register shifts and the linguistic demands of academic prose.

## Populations

Diglossia and orthographic complexity affect learners differently, with vulnerable groups experiencing their combined impact more severely. These populations show distinct processing profiles that reveal how multiplicative the challenges can be.

### Diglossic Challenges in Developmental Language Disorder

Children with developmental language disorder (DLD) are particularly sensitive to diglossic burdens. Saiegh-Haddad and Ghawi-Dakwar (2017) found that, although both DLD and typically developing groups struggled with the SpA–StA divide, the disadvantage was far greater for children with DLD. Difficulties were most pronounced for words containing phonemes or syllable structures unique to Standard Arabic, even when memory demands were controlled. These results suggest that children with DLD develop weaker and less accessible phonological representations of StA than their peers.

Recent work by Ghawi-Dakwar and Saiegh-Haddad (2025) confirms that children with developmental language disorder (DLD) face amplified diglossic challenges. Both DLD and typically developing children learned identical and cognate words more easily than Standard Arabic–unique forms, but the gap was far greater for the DLD group. For them, distant forms were especially difficult to acquire and retain. These findings show that diglossic distance limits not only phonological processing but also the formation of stable lexical representations. In DLD, linguistic distance and developmental vulnerability intersect, producing lasting disadvantages that require targeted interventions to reinforce core language abilities and support the transition between spoken and standard varieties.

### Orthographic Processing Deficits in Dyslexia

Children with dyslexia show distinct visual–orthographic difficulties that differ qualitatively from those of typically developing peers. Maroun et al. (2019) found that in tasks involving homograph resolution, orthographic parsing, and lexical decision, dyslexic readers performed well below age-matched controls and often resembled younger typical readers. These results suggest a developmental delay in the consolidation of orthographic processing skills essential for fluent reading.

These deficits extend beyond phonological weakness, revealing a dual vulnerability in Arabic dyslexia: impaired phonological awareness coupled with reduced sensitivity to visual–orthographic patterns. Khateb et al. (2013) showed that both skilled and dyslexic adult readers performed better with fully connected words than with nonconnected ones, yet dyslexic readers remained consistently slower and less accurate. This enduring gap underscores that orthographic difficulties in dyslexia persist despite experience and are not easily remediated.

What emerges from these findings is Arabic dyslexia involves more than phonological or general orthographic weakness. The cursive script itself adds a persistent burden, sensitivity to connectivity develops incompletely, leaving traces of difficulty even in adulthood. Dyslexia in Arabic is therefore a compound condition in which phonological and visual-orthographic deficits interact. Effective intervention must target both, reinforcing phonological awareness while explicitly training readers to manage script-specific features such as connectivity.

### The Double Burden: Combined Effects in Dyslexia

Research on dyslexia reveals how diglossic distance and orthographic opacity interact to compound reading difficulties. Schiff and Saiegh-Haddad (2016) compared dyslexic and typically developing pupils in Grades 2, 4, and 6 on word and pseudoword reading with and without vowelization. While typical readers improved with diacritics, dyslexic children showed no such benefit—their accuracy and fluency for Standard Arabic words remained low in both conditions. This indicates that vowelization, which normally eases orthographic processing, fails to mitigate the compounded challenges faced by dyslexic readers.

The disadvantage was greatest for Standard Arabic–unique words, showing that phonological deficits in dyslexia and diglossic distance reinforce each other to create a “double burden.” For these readers, the challenges of diglossia and orthographic opacity interact, altering the reading process itself. Consequently, supports such as vowelization that aid typical learners offer little relief. Effective intervention must therefore target both dimensions simultaneously, bridging phonological and morphological gaps while addressing script-specific obstacles linked to orthographic opacity.

## Discussion

This review synthesized research conducted over the past 25 years to examine the layered difficulties Arabic-speaking learners encounter in acquiring literacy. Four central themes emerged. First, the profound effect of diglossia and the linguistic distance between SpA and StA. Second, the impact of Arabic’s orthographic complexity on the development of core reading skills. Third, and the most distinctive contribution of this review, is the demonstration that these two domains do not operate separately but in a multiplicative manner, producing compounded challenges across development. Finally, the review highlights how these difficulties are distributed unevenly across learner populations, with vulnerable groups displaying qualitatively different processing patterns compared to typically developing peers.

In this review, a brief clarification of the term multiplicative is warranted. Multiplicative effects are not used in a formal statistical sense, nor simply as a synonym for interaction. Rather, the term is employed conceptually to describe situations in which diglossia and orthographic complexity mutually intensify one another, such that the impact of each constraint is qualitatively altered in the presence of the other. In contrast to additive effects, where difficulties accumulate linearly, or interaction effects understood narrowly as conditional influences, the multiplicative perspective highlights a compounding dynamic: linguistic distance increases the processing cost of an already opaque script, while orthographic opacity, in turn, amplifies the burden of unfamiliar linguistic forms. This framing helps explain why reading difficulties in Arabic are often disproportionate, persistent, and especially pronounced among vulnerable learners, even without invoking formal modeling.

Evidence consistently demonstrates that the diglossic divide exerts systematic influence on literacy outcomes. The linguistic distance between SpA and StA imposes persistent constraints on phonological awareness, word recognition, and reading comprehension, particularly in the earliest years of schooling (Asadi & Abu-Rabia, 2021; Khamis-Dakwar & Froud, 2007; Saiegh-Haddad, 2003). Preschoolers and first graders, for example, show markedly weaker performance when tasks involve StA-specific structures, and such difficulties extend into more advanced reading tasks across the school years. Findings from lexical matching paradigms corroborate this trend: children perform with greater accuracy and speed on identical forms shared by both SpA and StA than on StA-unique forms, with the performance gap widening as literacy demands grow (Asadi & Asli-Badarneh , 2023; Saiegh-Haddad & Joshi, 2014).

Research on orthography adds further complexity. Beginning readers struggle with positional allography and visually similar letters, while cursive connectivity initially hinders but later facilitates fluency. Morphological processing, in contrast, imposes persistent demands (Abu-Rabia, 2007; Asaad & Eviatar, 2013; Khateb et al., 2014; Taha, 2013). Crucially, diglossic and orthographic challenges amplify one another. Their interaction is most evident in contexts of unfamiliar vocabulary, syntactic complexity, or unvoweled text, where children must rely on morphology and context to compensate (Asaad & Eviatar, 2013; Saiegh-Haddad & Henkin-Roitfarb, 2014). This evidence strengthens the claim that difficulties in Arabic are not additive but multiply to create disproportionate burdens for certain groups.

Thus, literacy acquisition in Arabic must be understood as a developmental process shaped by the dynamic interplay of sociolinguistic and orthographic pressures. Learners with developmental or language difficulties are particularly susceptible, and their profiles illustrate the compounded nature of these challenges (Bergstrand Othman, 2025; Saiegh-Haddad & Ghawi-Dakwar, 2017; Saiegh-Haddad & Schiff, 2016). Existing theoretical models, including Arabic-specific ones, fall short because they treat diglossia and orthography as parallel variables rather than as dynamically interacting forces. (Saiegh-Haddad, 2017).

## Comparative Perspective on Literature

Across studies, there is broad agreement that diglossia exerts a lasting influence on literacy development, though the weight of its impact shifts with age and linguistic domain. Foundational work by Saiegh-Haddad (2003, 2005) and more recent studies (Asadi & Asli-Badarnehet al., 2023) demonstrate that the linguistic distance between SpA and StA is not confined to phonological awareness. Rather, it extends to morphology, vocabulary, and syntax (Abu-Rabia et al., 2022; Asadi et al., 2024; Shahbati-Kassem et al., 2025), thereby constraining both basic reading skills and higher-order comprehension (Asli-Badarneh & Asadi, 2023; Saiegh-Haddad, 2005). The pattern is consistent: the greater the departure from everyday SpA forms, the greater the processing cost. Corpus analyses underscore this reality, showing that only a small fraction of children’s lexicon consists of identical SpA–StA forms (Saiegh-Haddad & Spolsky, 2014).

Still, the effect is not uniform. Teachers in Saudi preschools, for instance, report orthography as more problematic than diglossia (Hashem, 2022). Other findings suggest that StA can sometimes support processing—for example, in deletion tasks (Asadi & Ibrahim, 2014) or phoneme isolation predicting fluency (Saiegh-Haddad, 2005). Longitudinal evidence points to partial adaptation: disadvantages ease somewhat in later grades, though morphology remains a bottleneck (Saiegh-Haddad & Haj, 2018; Schiff & Saiegh-Haddad, 2018). These mixed results illustrate that the “diglossic effect” cannot be treated as a single entity; it varies across domains, tasks, and developmental stages.

Evidence on orthography highlights letter position, connectivity, and diacritics as central constraints (Khateb et al., 2014; Taha, 2013). For skilled readers, connectivity supports fluency, while struggling readers continue to experience it as a barrier (Saiegh-Haddad & Schiff, 2016). Vowelization helps beginners but loses importance with age. What remains underexplored is the *interaction* of diglossia and orthography. Studies show that orthographic load is heavier for StA-unique items, especially for children with weaker phonological or morphological skills (Asaad & Eviatar, 2013). Vulnerable populations show the clearest evidence of this “double challenge” (Saiegh-Haddad & Ghawi-Dakwar, 2017; Saiegh-Haddad & Schiff, 2016). This reinforces the central claim of the present review: Arabic literacy difficulties are best understood as compounded, multiplicative effects.

What has received far less systematic attention, however, is the interaction between these two domains. Evidence suggests that orthographic complexity weighs most heavily on items from the StA variety, particularly for learners with weaker phonological or morphological skills (Asaad & Eviatar, 2013). Population studies reinforce this point: children with learning difficulties are disproportionately affected by the combined burden of diglossia and orthography. Their vulnerabilities in phonological awareness and morphological processing become amplified under this “double challenge,” leaving them at higher risk for persistent reading and academic difficulties (Bergstrand Othman, 2025; Saiegh-Haddad & Ghawi-Dakwar, 2017; Saiegh-Haddad & Schiff, 2016). This body of evidence highlights the central argument of the present review—that the effects of diglossia and orthographic complexity cannot be understood in isolation but must be conceptualized as multiplicative in nature.

## Challenging and Extending Theoretical Perspectives

It is important to clarify that the present review does not argue that widely used models of reading are inadequate in a general sense. Rather, the critique advanced here concerns the scope and boundary conditions of these models when applied to Arabic literacy. Specifically, several core assumptions embedded in dominant frameworks-such as relative continuity between spoken and written language, early transparency of grapheme-phoneme correspondences, and stable mappings between phonological, orthographic, and lexical representations-are systematically strained in the case of Arabic. In diglossic contexts, the linguistic distance between SpA and StA challenges assumptions of continuity between oral language and print. At the same time, the visual and orthographic characteristics of the Arabic script complicate assumptions about transparency and incremental decoding. Accordingly, existing theoretical models were evaluated in this review not in terms of their internal coherence, but in terms of their explanatory adequacy in accounting for empirical patterns repeatedly observed in Arabic literacy research-namely, whether they can explain the diglossic gap, orthographic constraints, and the interaction of these factors across developmental stages and learner populations. This empirically grounded perspective highlights that while dominant models capture important components of reading, they offer an incomplete account of how these components interact and unfold developmentally in Arabic.

The body of evidence reviewed here calls for a reconsideration of how Arabic literacy is framed theoretically. Dominant models such as the Simple View of Reading (SVR), the dual-route model, and the Lexical Quality Hypothesis (LQH) rest on assumptions of stable and transparent mappings between oral and written forms. These assumptions, developed primarily on the basis of European languages, do not hold in Arabic, where the language of print is structurally distant from the (oral) language of daily speech and where the script imposes unique orthographic demands (Coltheart et al., 1993; Gough & Tunmer, 1986; Perfetti, 2007).

In particular, the SVR’s reliance on oral language comprehension as the foundation for reading is challenged by the fact that many of the linguistic forms children must decode in StA have no equivalent in their SpA repertoire. The LQH presupposes the development of unified, high-quality lexical representations, yet in Arabic these representations are divided across two language varieties with differing phonological, morphological, and lexical realizations. Arabic-specific frameworks, such as the MAWRID model (Saiegh-Haddad, 2017), represents a central and well-established Arabic-specific model of reading development, making an important contribution by explicitly integrating vowelization, morphological structure, and diglossic distance into an account of word reading in Arabic. Substantial empirical evidence reviewed in this manuscript supports MAWRID’s core assumptions regarding early decoding mechanisms, the role of morphology, and the influence of linguistic distance between spoken and standard Arabic on word recognition processes. At the same time, the present review suggests that certain phenomena-such as cross-dialectal variation, the developmental transition from vowelized to unvowelized script, and the emergence of qualitatively distinct reading trajectories among learners with reading or language difficulties-are not yet fully articulated within the current scope of the model. These gaps reflect limitations of coverage rather than conceptual inadequacy, and point to opportunities for further theoretical elaboration aimed at capturing the dynamic interaction between diglossia and orthographic complexity across development.

The cumulative evidence therefore challenges the very assumptions on which these models are built. It suggests that diglossia and orthographic complexity cannot be treated as independent variables, but as interacting forces whose multiplicative effects alter developmental pathways. Moreover, the Arabic case does not only call for a “local” model; it exposes blind spots in supposedly universal theories of reading, showing that without incorporating Arabic evidence, global literacy frameworks remain incomplete. This reconceptualization requires a framework that is not only script- and language-sensitive but also developmentally and educationally grounded (Eviatar & Ibrahim, 2014; Saiegh-Haddad & Henkin-Roitfarb, 2014).

While the review does not propose a new formal model of reading, it advances a more comprehensive conceptual perspective for understanding Arabic literacy. This perspective emphasizes that diglossia and orthographic complexity should be conceptualized as interacting and multiplicative constraints, rather than as parallel or additive sources of difficulty. By synthesizing evidence across domains and populations, the review points to the need for theoretical accounts that integrate linguistic distance, script-specific constraints, and developmental variability within a single explanatory framework. In this sense, the contribution of the review lies in strengthening existing models (both universal and Arabic-specific) by refining their underlying assumptions and improving their alignment with the empirical realities of Arabic literacy development.

It is important to note that the effects described in this review should not be interpreted as uniform across all learners or educational contexts. Although common patterns emerge across studies, the impact of diglossia and orthographic complexity is likely to vary as a function of learner background, language experience, instructional context, and access to linguistic and educational resources. Differences related to dialectal exposure, socioeconomic conditions, and educational support may shape how these constraints are experienced and managed across development.

## Theoretical and Pedagogical Implications

The evidence reviewed makes clear that progress in theorizing Arabic literacy requires a framework that is both context-sensitive and multi-layered. Such a framework must account for how diglossia and orthographic complexity operate not as isolated variables but as interdependent factors that shape literacy trajectories in a multiplicative fashion. It must also capture how these effects shift with age, task demands, and learner profile (Asaad & Eviatar, 2013; Saiegh-Haddad & Henkin-Roitfarb, 2014). This point is crucial, since the acquisition of academic literacy in Arabic relies simultaneously on oral skills in Standard Arabic and on decoding skills in a script that is morphologically dense and visually complex. The combined load of these demands is not explained by current linear models and requires theoretical reconceptualization (Khamis-Dakwar & Froud, 2007; Saiegh-Haddad & Henkin-Roitfarb, 2014). Models must also take into account children with language and learning difficulties, whose developmental pathways provide clear evidence that challenges accumulate in an amplifying, rather than additive, manner.

From a pedagogical standpoint, the implications are equally significant. Instruction must be designed to systematically bridge the gap between spoken and standard varieties of Arabic, with explicit scaffolding at both sublexical levels (phonology, morphology) and the lexical level (vocabulary). The transition from vowelized to unvowelized script constitutes a critical developmental juncture that requires careful pedagogical management, rather than being left to incidental exposure (Abu-Rabia & Hijjazi, 2020). Moreover, root–pattern morphology, positional allography, and letter connectivity should not be reserved for advanced literacy instruction but treated as foundational components of early reading curricula (Khateb et al., 2014; Taha, 2013).

Equally important are the broader educational stakes. Persistent gaps in international assessments such as PIRLS reflect the compounded barriers of diglossia and orthography (Saiegh-Haddad & Joshi, 2014). The persistent performance gaps observed in PIRLS should be interpreted within a broader systemic context. Reading achievement is shaped by multiple external factors, including socioeconomic conditions, instructional quality, access to print, and language exposure outside school. The present review does not suggest that diglossia and orthographic complexity operate in isolation or constitute the sole explanation for these gaps. Rather, these linguistic characteristics are best understood as structural constraints that interact with broader systemic factors. For Arabic-speaking learners, acquiring literacy in a language variety that differs substantially from everyday speech, combined with the visual and orthographic complexity of the script, places additional cognitive and instructional demands on learners and educators. These demands may amplify the effects of external risk factors, particularly in contexts with limited educational resources or reduced exposure to StA, contributing to performance gaps that tend to persist despite educational reforms. This underscores that the issue is not only cognitive but also systemic: without targeted interventions, Arabic-speaking learners are at risk of entrenched educational inequality. Intervention programs must reflect the compounded reality of Arabic literacy. They should be tailored to the sociolinguistic context, sensitive to dialectal diversity, and responsive to the needs of vulnerable populations (Saiegh-Haddad & Ghawi-Dakwar, 2017; Saiegh-Haddad & Schiff, 2016). Importantly, such programs must address higher-order comprehension skills alongside basic decoding processes, since both are simultaneously shaped by the interaction of diglossic and orthographic pressures (Saiegh-Haddad & Joshi, 2014). Only pedagogies that directly engage with this compounded challenge can effectively support learners across the full spectrum of ability and context.

## Limitations and Directions for Future Research

### Limitations of Universal Models

Universal reading models such as the SVR, the Dual Route Model (Coltheart et al., 1993), and the Lexical Quality Hypothesis (Perfetti, 2007) face clear limits when applied to Arabic. Developed for languages with close alignment between speech and print, these models overlook the diglossic gap in Arabic, where children acquire the spoken vernacular but learn literacy in Standard Arabic. The Dual Route Model assumes stable grapheme–phoneme mapping, yet Arabic’s positional letter forms, cursive connectivity, and optional vowelization disrupt this stability. Likewise, the Lexical Quality Hypothesis presumes independent phonological, orthographic, and semantic systems, while in Arabic these are intertwined and shaped by diglossic distance and script complexity. These factors constrain the explanatory reach of existing models and highlight the need for frameworks attuned to Arabic’s linguistic and orthographic structure.

Despite progress, research on Arabic literacy remains narrow in scope and design. Most evidence comes from controlled tasks using isolated words or pseudowords, which clarify specific processes but lack ecological validity and fail to reflect comprehension in connected or classroom contexts. Longitudinal data are also missing, leaving unclear how children adjust from vowelized to unvoweled script or how diglossic distance changes as vocabularies grow. Geographically, research is concentrated in a few national and dialectal settings, limiting generalizability. Broader, comparative studies across dialects and educational contexts are still largely absent.

Future work should emphasize longitudinal and classroom-based studies that follow how children adapt to diglossic distance and orthographic transition, focusing on comprehension, discourse, and higher-level literacy skills. Research must also include learners with language and reading difficulties, whose experiences reveal the compounded nature of these challenges and guide targeted pedagogy.

Progress further depends on developing and validating integrative theoretical frameworks. The current focus on isolated tasks and grade-specific samples limits coherence. A unified model should explain how diglossia and orthographic complexity interact with development, sociolinguistic context, and learner diversity to capture Arabic literacy in its full complexity.

## Summary

Across the four research questions, Arabic literacy is shaped by interacting, not additive, challenges. The linguistic divide between spoken and standard varieties constrains core reading processes, while the orthographic system adds shifting visual and morphological demands. These factors combine to produce varied effects across tasks, developmental stages, and learner profiles, with vulnerable groups most impacted. Arabic literacy thus requires integrative models that link linguistic and orthographic dimensions and teaching approaches that bridge varieties and script. By revealing these multiplicative effects, Arabic serves as both a test and a refinement ground for universal reading theories.

## Data Availability

The data that support the findings of this study are available from the corresponding author upon request.

## Appendix

See Table 1.

**Table 1:** Summary of reviewed studies on Arabic diglossia and orthography (2000–2025)

Study	Participants	Focus	Task	Findings	Diglossia/orthography
Saiegh-Haddad (2003)	N = 30 K–Gr1	Phonological distance (SpA vs. StA)	Phoneme isolation; pseudoword decoding	StA-only phonemes/syllables hardest; shared categories spared	Diglossia
Saiegh-Haddad (2018)	N = 120 K, Grade 1, 2, 6	Phonological distance (SpA ↔ StA) → quality of phonological representations	Picture-supported pronunciation-accuracy judgment on StA words (identical, cognate: 1-vowel/1-consonant/2-phoneme/ > 2-phoneme, unique); mixed-effects analysis	Greater SpA–StA distance → weaker phonological representations across grades; identical > cognates (consonant/2 +) ≈ unique; 1-vowel cognates ≈ identical;	Diglossia
Asadi & Abu-Rabia (2021)	N = 261, K → Gr1	Lexical distance; PA & RAN	PA, RAN with SpA/StA/shared/pseudowords	Spoken > StA/unique; length > position; RAN slower for StA	Diglossia
Asadi & Ibrahim (2014)	N = 41, Grade 1	Phonological awareness under diglossia	Phoneme deletion (SpA vs. StA words)	Better performance for StA items in deletion tasks, suggesting StA can sometimes support processing	Diglossia (phonology)
Saiegh-Haddad and Haj (2018)	N = 120 K, Grade 1, 2, 6	Phonological distance and representations	Pronunciation accuracy judgment	Phonological distance significantly affected representational quality across all grades; identical > cognates > unique; effect persisted but lessened in Grade 6	Diglossia (phonological representations)
Khamis-Dakwar and Froud (2007)	5 adult native speakers of Palestinian Arabic	Neurocognitive processing of diglossia	ERP study: sentence reading in SpA and StA, with code-switches and semantic anomalies	Code-switching between SpA and StA elicited a P600 similar to bilingual switching; semantic anomalies in StA triggered stronger N400 effects; results show SpA and StA are processed as distinct linguistic systems	Diglossia
Tarabeh et al. (2025)	N = 41 Grade 1	Diglossic effects on phonological working memory	Verbal retrieval and remembering instructions tasks in SpA vs. StA	Better performance in verbal retrieval tasks with SpA stimuli; no significant differences in remembering instructions; increased processing demands highlighted diglossic load on working memory	Diglossia (phonological WM)
Asli-Badarneh & Asadi (2023)	N = 180 Gr1–2	Lexical distance (identical/cognate/unique)	Word & text reading	Stable gradient: identical > cognate > unique; gaps widen by grade	Diglossia
Shahbari-Kassem et al. (2024)	N = 80 Kindergarten	Diglossic effects on listening comprehension and vocabulary	Scaffolded storybook reading in SpA vs. StA (identical/cognate/unique forms)	Comprehension and vocabulary highest in SpA stories; performance declined as StA share increased; unique StA forms hardest; scaffolded exposure partially supported learning	Diglossia (LC, vocabulary)
Abu-Rabia (2000)	N = 282 Gr1–2	Diglossia and comprehension	Story comprehension (explicit/inferential)	Children with less exposure to StA worse; inferential hardest	Diglossia
Joubran-Awadie and Shalhoub-Awwad (2023)	(analytic) N = 480 morphemes	Morphological distance SpA–StA	Structural mapping	> 80% morphemes differ; especially verb inflections	Diglossia (morphology)
Asadi et al. (2024)	N = 261 Kindergarten → Grade 1 (longitudinal)	Morphological diglossic distance (gender, number, possessive, tense)	Morphological awareness tasks (SpA vs StA inflections); reading, spelling, comprehension	Children performed better on SpA than StA across inflections; difficulty order: gender > number > possessive > tense; morphological knowledge in K predicted G1 spelling, reading fluency, and comprehension; StA contributed an additional 2–3% variance beyond SpA	Diglossia (morphology)
Abu-Rabia, Sarsour Kedan, & Wattad (2022)	N = 63 Grade 4 (regular vs. struggling readers)	Diglossic effects on syntax	Tasks assessing mastery of Standard Arabic vs. spoken-syntax structures	Mastery of StA syntactic structures developed more slowly and less consistently than overlapping spoken forms; results show diglossia constrains syntactic competence	Diglossia (syntax)
Khateb et al. (2014)	N = 90 Gr3, 6, 9	Cursive connectivity (development)	Lexical decision with connectivity manipulations	Gr3: connected hardest; Gr6: connected > nonconnected; Gr9: effect fades	Orthography
Abdelhadi et al. (2011)	N = 60 Gr3 & Gr6 (Arabic/Hebrew)	Visual complexity; connectivity	Vowel detection	Hebrew faster; in Arabic connected > disconnected; no word superiority in Gr6	Orthography
Yassin et al. (2020)	N = 96 Gr1,2,4	Visual-orthographic errors	Spelling analysis	Visual-orthographic errors > ¼ of errors; ligaturing & confusions common	Orthography
Abu-Rabia & Hijjazi (2020)	N = 480 Gr5,7,9	Vowelization × genre	Text comprehension	Vowelization helps Gr5 most; genre dependent	Orthography
Taha and Azaizah-Seh (2017)	N = 41 university	Vowelization in skilled readers	Lexical decision	Adults faster & more accurate on unvoweled words	Orthography
Abu-Rabia (2007)	N = 80 Gr3/6/9/12; typical & dyslexic	Morphological awareness	Morphological ID/production; reading	Morphology predicts word reading & comprehension	Orthography (morphology)
Saiegh-Haddad and Schiff (2016)	N = 100 Gr2–10	Variety distance; vowelization	Word & pseudoword reading (vowelized/unvowelized)	StA < SpA across accuracy/fluency; effects persist; diacritics partial relief	Diglossia + Orthography
Asaad and Eviatar (2013)	N = 60 Gr1,3, adults	Orthographic distortion × development	Stroop; rapid naming	By Gr3, greater interference for correct vs distorted words; RAN slowed by StA sounds	Diglossia + Orthography
Saiegh-Haddad (2005)	N = 30 Gr1 (vowelized)	Early reading: PA/RAN/WM → fluency	Pseudoword fluency; phoneme isolation; letter recoding	Letter recoding drives fluency; StA phonemes harder	Diglossia + Orthography
Saiegh-Haddad and Schiff (2025)	N = 112 Gr3	SpA skills → StA comprehension	Morphological awareness, vocab, decoding, listening	SpA morph-awareness strongest predictor; explains ~ 41% variance	Diglossia + Orthography
Abu-Liel et al. (2021)	N = 77 Gr8	Script & genre	Reading in vowelized/unvowelized MSA, Arabizi	Unvoweled fastest; comprehension higher with vowels; genre effect	Diglossia + Orthography
Saiegh-Haddad and Ghawi-Dakwar (2017)	N = 100 Children with DLD vs controls	Diglossia in DLD	Word & nonword repetition	DLD children show amplified diglossic disadvantage	Populations (DLD) + Diglossia
Ghawi-Dakwar and Saiegh-Haddad (2025)	N = 100 K/Gr1; TLD & DLD	Phonological distance effect on word learning	Word learning tasks (SpA vs StA forms)	All children worse with StA-distant forms; effect stronger in DLD	Populations (DLD) + Diglossia
Maroun et al. (2019)	N = 60 Dyslexic vs typical children	Orthographic processing in dyslexia	Homograph resolution; parsing; lexical decision	Dyslexics lag and resemble younger readers; dual vulnerability	Populations (Dyslexia) + Orthography
Khateb et al. (2013)	N = 40 Adult skilled vs dyslexic	Connectivity in dyslexia	Lexical decision with connectivity	Fully connected > nonconnected; dyslexics less efficient overall	Populations (Dyslexia) + Orthography
Schiff and Saiegh-Haddad (2017)	N = 60 Gr2,4,6; dyslexic vs controls	Double burden: distance × vowelization	Word/pseudoword reading (± diacritics)	Vowelization helps typical but not dyslexics; StA-unique hardest	Interaction + Populations
